# Identification of New Angiotensin-Converting Enzyme Inhibitory Peptides Isolated from the Hydrolysate of the Venom of *Nemopilema nomurai* Jellyfish

**DOI:** 10.3390/toxins16090410

**Published:** 2024-09-20

**Authors:** Ramachandran Loganathan Mohan Prakash, Deva Asirvatham Ravi, Du Hyeon Hwang, Changkeun Kang, Euikyung Kim

**Affiliations:** 1 College of Veterinary Medicine, Gyeongsang National University, Jinju 52828, Republic of Korea; mohan_22@gnu.ac.kr (R.L.M.P.); devabiochem@gnu.ac.kr (D.A.R.); pooh9922@hanmail.net (D.H.H.); ckkang@gnu.ac.kr (C.K.); 2 Institute of Animal Medicine, Gyeongsang National University, Jinju 52828, Republic of Korea

**Keywords:** *Neophilia nomurai*, papain enzyme hydrolysate, chromatography, angiotensin-converting enzyme (ACE) inhibitor, peptide identification

## Abstract

Recently, jellyfish venom has gained attention as a promising reservoir of pharmacologically active compounds, with potential applications in new drug development. In this investigation, novel peptides, isolated from the hydrolysates of *Nemopilema nomurai* jellyfish venom (NnV), demonstrate potent inhibitory activities against angiotensin-converting enzyme (ACE). Proteolytic enzymes—specifically, papain and protamex—were utilized for the hydrolysis under optimized enzymatic conditions, determined by assessing the degree of hydrolysis through the ninhydrin test. Comparative analyses revealed that papain treatment exhibited a notably higher degree of NnV hydrolysis compared to protamex treatment. ACE inhibitory activity was quantified using ACE kit-WST, indicating a substantial inhibitory effect of 76.31% for the papain-digested NnV crude hydrolysate, which was validated by captopril as a positive control. The separation of the NnV-hydrolysate using DEAE sepharose weak-anion-exchange chromatography revealed nine peaks under a 0–1 M NaCl stepwise gradient, with peak no. 3 displaying the highest ACE inhibition of 96%. The further purification of peak no. 3 through ODS-C18 column reverse-phase high-performance liquid chromatography resulted in five sub-peaks (3.1, 3.2, 3.3, 3.4, and 3.5), among which 3.2 exhibited the most significant inhibitory activity of 95.74%. The subsequent analysis of the active peak (3.2) using MALDI–TOF/MS identified two peptides with distinct molecular weights of 896.48 and 1227.651. The peptide sequence determined by MS/MS analysis revealed them as IVGRPLANG and IGDEPRHQYL. The docking studies of the two ACE-inhibitory peptides for ACE molecule demonstrated a binding affinity of −51.4 ± 2.5 and −62.3 ± 3.3 using the HADDOCK scoring function.

## 1. Introduction

Animal venoms are the intricate blends of various pharmacologically active elements, encompassing proteins, peptides, and enzymes with specific biological functions related to as-yet-unidentified substances [1,2]. Based on a report in 2020 [3], there are several drugs that have been identified from animal toxins, such as captopril, enalapril (ACE inhibitors; Jararaca pit viper; US drug administration 2020), exenatide (type 2 diabetes mellitus; Gila monster lizard; US drug administration 2020), Tirofiban (acute coronary syndrome; saw-scaled viper; US drug administration 2020), etc.

Hypertension (BP) refers to significant risk factors that contribute to cardiovascular disease (CVD) [4]. It is a worldwide health issue, affecting over 1.3 billion people with elevated blood pressure across the globe [5]. Aiming for one of the global targets on non-communicable diseases (NCDs) set by the World Health Assembly in 2013, there is a goal to reduce the prevalence of elevated blood pressure by 25% by 2025, as compared to its level in 2010. According to self-reported data from a survey on hypertension prevalence involving 533,306 adults, eradicating hypertension in women would reduce population mortality by approximately 7.3% compared with 0.1% for hyperlipidemia, 4.1% for diabetes, 4.4% for cigarette smoking, and 1.7% for obesity. Conversely, in men, eradicating hypertension would decrease population mortality by approximately 3.8%, compared to 2.0% for hyperlipidemia, 1.7% for diabetes, 5.1% for cigarette smoking, and 2.6% for obesity [6]. Elevated blood pressure is characterized by a systolic blood pressure (SBP) of ≥140 mmHg or diastolic blood pressure (DBP) of ≥90 mmHg [7,8]. The first-line treatments for hypertension consist of beta-blockers, angiotensin-converting enzyme (ACE) inhibitors, angiotensin II receptor blockers (ARBs), loop and thiazide diuretics, and dihydropyridine calcium channel blockers (CCBs) [9]. Among these, ACE inhibitors constitute a significant class of drugs. ACE inhibitors are prescribed for uncomplicated hypertension and are also recommended for conditions such as hypertension with concurrent CAD (including post-myocardial infarction), chronic kidney disease (CKD), type 2 diabetes, heart failure with reduced ejection fraction, or atrial fibrillation [10]. 

Jellyfish have thrived in areas where numerous other species have faced decline. The adaptations of these species, which have been around for 500 million years, are so effective that they now significantly influence human activities. Nematocyte, a unique type of cell found in jellyfish, possesses stinging capabilities and is utilized for defense, prey capture, and locomotion. These specialized cells consist of a thread-like aperture that resembles an anchor, serving as a reservoir for jellyfish venom. Jellyfish venom causes swelling, which leads to a rapid heart rate, difficulty in respiration, severe-to-mild back pain, and brain hemorrhages [11]. On the other hand, jellyfish venom also possesses many pharmacological activities in addition to its toxic effects. For example, *Cyanea capillata*—anti-arrhythmic activity [12]; *Chiropsalmus quadrigatus*—anti-hypertensive activity [13]; *Rhopilema esculentum*—immunomodulatory activity [14]; *Chrysaora quinquecirrha* [15]—anti-inflammatory, anti-microbial, and analgesic activity; *Pelagia noctiluca*—anti-inflammatory activity [16]; and *Carybdea marsupialis*—anti-microbial activity [17].

*Nemopilema nomurai*, a rhizostome jellyfish within the phylum Cnidaria, holds the distinction of being one of the world’s largest jellyfish, featuring a bell diameter of 2 m and a body weight reaching up to 200 kg [18]. In recent times, there has been a proliferation of jellyfish species, including *N. nomurai*, in the coastal waters of East Asia [19]. NnV harbors diverse pharmacological characteristics such as anti-inflammatory and anti-cancer properties [20,21,22,23]. Previously, it has been reported that whole *Nemopilema nomurai* jellyfish proteolytic hydrolysate shows ACE-inhibitory activity [24]. Therefore, in this study, we specifically aim for the identification of ACE-inhibitory peptides from *Nemopilema nomurai* jellyfish venom (NnV) using papain hydrolysate.

## 2. Results

### 2.1. Optimization of Enzymatic Condition upon NnV Using the Degree of Hydrolysis

**Figure 1:**
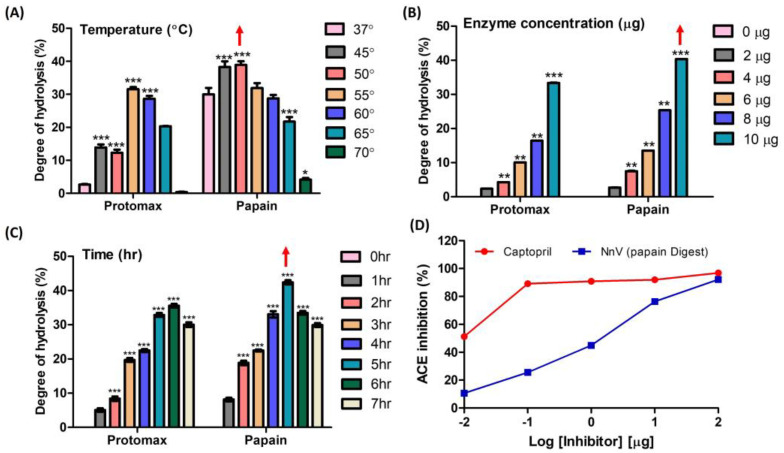
Degree of enzyme hydrolysis upon NnV based on the Ninhydrin test and preliminary ACE inhibition. (**A**) Effect of different temperatures of enzyme incubation upon NnV; (**B**) Effect of enzyme concentrations on NnV; (**C**) Effect of reaction time of enzymes upon NnV; (**D**) Log-inhibitory curve of the percent ACE inhibition of papain-hydrolyzed NnV (positive control: captopril). Red arrow denotes highest hydrolysis condition. Results are expressed as mean ± standard deviation (SD), with significance denoted by * *p* < 0.05, ** *p* < 0.01, and *** *p* < 0.001.

Temperature, reaction time, and concentration play pivotal roles in the enzymatic hydrolysis of NnV. From ninhydrin colorimetry assay, the optimal conditions for papain and protomex were determined as follows: a temperature range of 45–50 °C (papain) and 55–60 °C (protomex), a concentration of 10 µg, and a reaction time of 5–6 h. These conditions yielded the highest degrees of hydrolysis, with values of 31.54%, 33.35% and 35.57% for protomex, and 38.8%, 40.37% and 42.4% for papain (Figure 1A–C). In Figure 1D, the wide range of hydrolysate concentrations were tested for their ACE-inhibitory activities to determine the optimum concentration for further study. For this, 100, 10, 1, 0.1, and 0.01 µg/20 µL of NnV–papain hydrolysate were examined to assess ACE-inhibitory activity. Among these, 10 µg/20 µL was chosen for further study, which shows a concentration-dependent inhibition in ACE assay (76.31% of maximum inhibitory effect) without reaching saturation range. Therefore, we considered a 10 µg/20 µL concentration of the hydrolysate as optimum for further investigation. The positive control, captopril, exhibited a 91.92% inhibition under the same conditions (Figure 1D).

### 2.2. Purification of ACE-Inhibitory Peptides Guided by In Vitro Assay

#### 2.2.1. Ion-Exchange Chromatography

**Figure 2:**
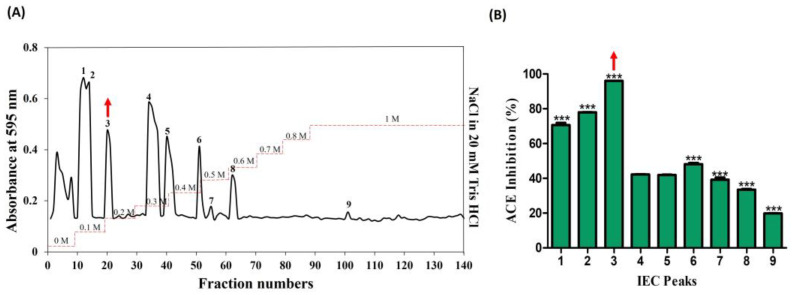
Purification of ACE-inhibitory fractions using DEAE sepharose anion-exchange chromatography. (**A**) Chromatogram of fractions that were isolated from hydrolyzed crude NnV. (**B**) ACE-inhibitory activity of separated fractions. The red arrows denote the active peak. Results are expressed as mean ± standard deviation (SD), with significance denoted by *** *p* < 0.001. The numbers 1–9 in (**A**) denote isolated peaks.

The venom from jellyfish, subjected to papain digestion under optimal conditions (50 °C; 5 h; 10 µg), was freeze-dried and reconstituted in a 20 mM Tris HCl solution (equilibrium buffer) for subsequent sequential chromatography. In the preliminary stage, ion-exchange chromatography revealed the presence of nine peaks, predominantly eluting within the range of 0–1 M NaCl in 20 mM Tris HCl (Figure 2A). The peaks were collected and lyophilized, and protein concentration was determined by protein assay. All the peaks were normalized and their ACE-inhibitory (%) activities corresponding to these nine peaks were determined as follows: 70.64, 77.69, 96.0, 42.21, 41.87, 48.14, 39.21, 33.48, and 19.75. Notably, among these, peak no. 3 demonstrated the highest activity, at 96% (Figure 2B).

#### 2.2.2. Reverse-Phase High-Pressure Liquid Chromatography

**Figure 3:**
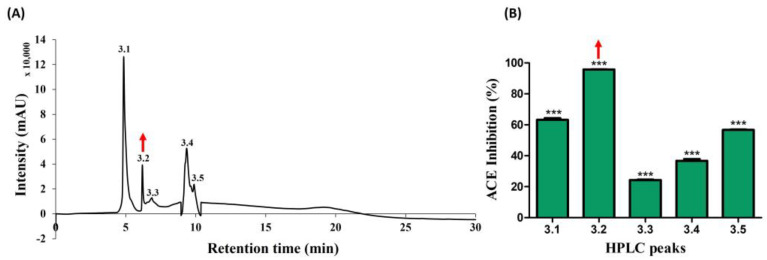
Further separation of ACE-inhibitory fractions using RP-HPLC. (**A**) Chromatogram of sub peaks from active peak no. 3 isolated (**B**) ACE-inhibitory activity. The red arrow marks denote the active peak. Results were expressed as mean ± standard deviation (SD), with significance denoted by *** *p* < 0.001. The numbers 3.1–3.5 in (**A**) denotes isolated peaks.

Subsequently, the active candidate (peak no. 3) underwent further purification through reverse-phase high-pressure liquid chromatography (RP-HPLC), employing an ODS-C18 column. Five distinct peaks (3.1, 3.2, 3.3, 3.4, and 3.5) were successfully isolated, as illustrated in Figure 3A, with retention times falling within the range of 5–10 min. Later, peaks were collected using a fraction collector, acetonitrile was removed using a decompression concentrator, and the ACE-inhibitory activities (%) were assessed based on protein concentration. The respective activities of the five peaks ranged from 20% to 96%, with values determined as follows: 65.19, 95.74, 24.57, 37.87, and 56.50 (shown in Figure 3B). A noteworthy observation was exhibited in peak no. 3.2 with 95.74%, mirroring the previously identified active peak. As a result, this potent candidate was chosen for further analysis using MALDI–TOF/MS.

### 2.3. MALDI–TOF/MS Examination

**Figure 4:**
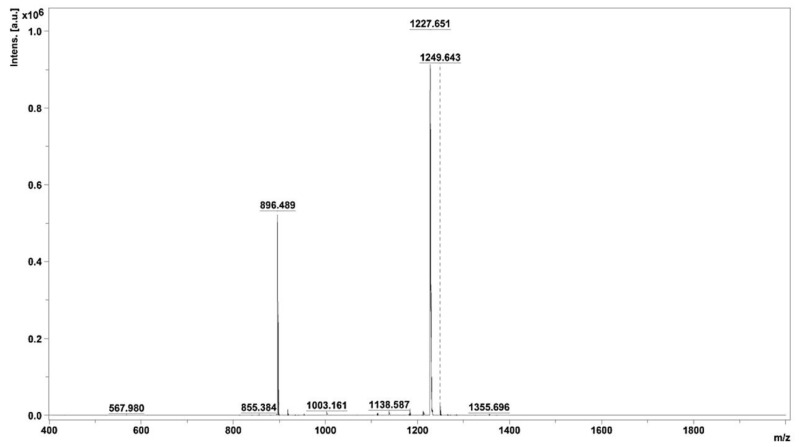
Mass spectrum of fraction 3.2, obtained using MALDI–TOF/MS.

Peak 3.2, displaying noteworthy activity, underwent comprehensive analysis utilizing MALDI–TOF/MS, thereby elucidating the identification of two distinct peptides characterized by varying molecular sizes. The average molecular masses of these identified peptides were meticulously determined, resulting in values of 896.489 and 1227.651, as visually represented in Figure 4. This analytical insight provides crucial information regarding the molecular composition of the peptides residing within active peak 3.2.

### 2.4. ACE-Inhibitory Peptides Sequencing Using MS/MS 

**Figure 5:**
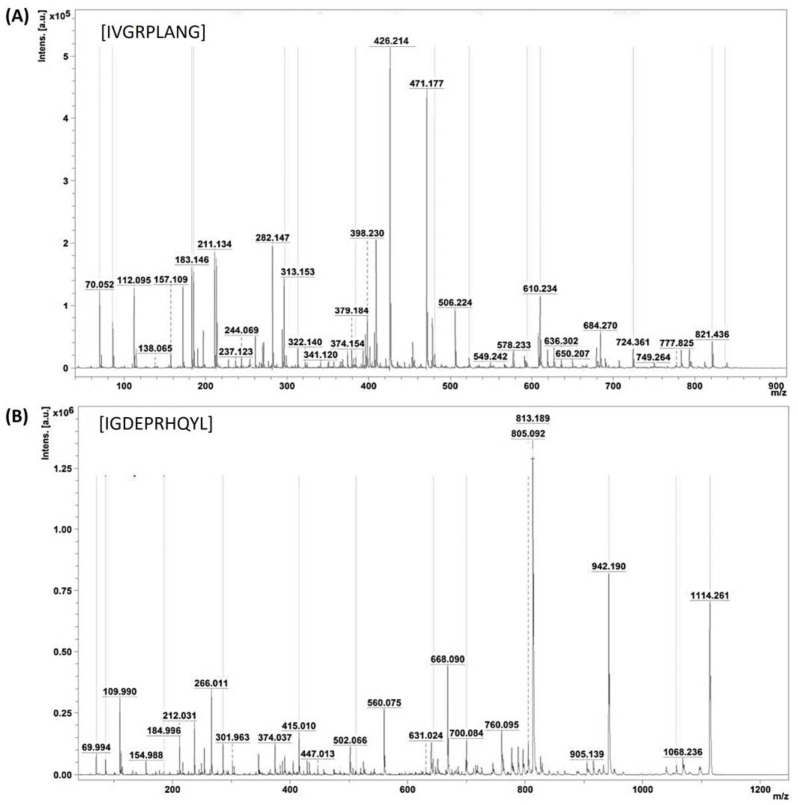
Identification of ACE-inhibitory peptide sequence. (**A**,**B**) MS/MS peaks of identified peptides IVGRPLANG and IGDEPRHQYL from NnV-hydrolysate.

Following the identification of the two peptides within active peak 3.2—namely, those with average molecular masses of 896.489 and 1227.651—an in-depth exploration of their amino acid sequences was undertaken. This involved a thorough analysis of the MS/MS ionic patterns, as illustrated in Figure 5A,B, utilizing the advanced capabilities of Brucker Flex analysis version 3.4 software. The intricacies of the peptides’ structural compositions, denoted as IVGRPLANG and IGDEPRHQYL, were thereby elucidated through the intricate patterns discerned during the MS/MS analysis. It is noteworthy that the laser conditions employed for this analytical procedure were meticulously set at 45% laser intensity, with each peptide subjected to 1000 shots for optimal resolution and accuracy. 

### 2.5. Peptide Structure Prediction and Protein–Ligand Interactions through a Computational Approach

AlphaFold generates five peptide structures for a single model. In this, the top-ranked structure was selected based on both the pLDDT and pTM score. The three-dimensional structures of two peptides were modeled using the AlphaFold2 Colab notebook, which is depicted in Figures S1 and S2. The pTM metric, ranging from 0 to 1, serves to assess peptide structure predictions by providing a 3D error measurement. Additionally, the per-residue confidence score is determined through the predicted local distance difference test (pLDDT) score, which spans from 0 to 100. Scores exceeding 90 indicate a high confidence level, while scores below 50 indicate a low confidence level. For the IGDEPRHQYL peptide structure, the pLDDT and pTM scores of the top-ranked model were 72.9 and 0.0461, respectively. Similarly, for the IVGRPLANG peptide structure, the corresponding scores for the top-ranked model were 73.8 and 0.04, respectively. The analysis of the pLDDT results indicates a low confidence in the linker region, suggesting potential flexibility in this region. Detailed information on pLDDT and pTM score are illustrated in Table S1.

**Figure 6:**
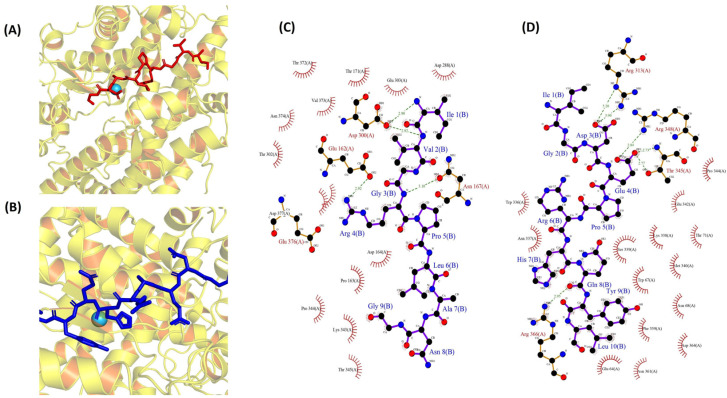
Molecular docking simulations of ACE (PDB: 1O8A) protein against the isolated peptides. (**A**,**B**) Depicting the optimal docking poses at the active site for IVGRPLANG (red) and IGDEPRHQYL (blue). (**C**,**D**) The peptides’ (IVGRPLANG and IGDEPRHQYL) interactions with ACE protein residues.

To investigate the molecular interactions between two peptides and ACE, a docking simulation was conducted, utilizing the flexible docking tool within the HADDOCK software (version 2.4). The docking investigation, involving the peptides IVGRPLANG and IGDEPRHQYL, revealed optimal poses (Figure 6A,B) with HADDOCK scores of −51.4 ± 2.5 (cluster 5) and −62.3 ± 3.3 (cluster 7), respectively. Detailed information regarding other interactions, along with their scores, is presented in Tables S2 and S3. In our study, the bonded interactions for IVGRPLANG against the ACE inhibitor involve GLU 162, ASN 167, ASP 300, ASP 300, ARG 4, ARG 4, ILE 1, and VAL 2, highlighted in red. Meanwhile, the non-bonded interactions include GLU 162, PRO 163, ASP 164, ASP 300, ASP 300, LYS 343, PRO 344, and THR 302, denoted by the black color in Figure 6C. The bonded interactions for IGDEPRHQYL against the ACE inhibitor encompass ARG 313, ARG 313, THR 345, THR 345, ARG 348, ARG 366, ASP 3, ASP 3, GLU 4, GLU 4, and GLN 8, highlighted in red. Conversely, the non-bonded interactions consist of GLU 64, TYR 9, LEU 10, TRP 67, LYS 338, SER 339, PRO 344, GLU 4, HIS 7, ARG 366, and MET 340, denoted by the black color in Figure 6D. The pivotal role of the Zn(II) ion in the mechanism of ACE inhibition necessitated the validation of its positioning through ligplot analysis. Following peptide docking, notable alterations in the position, bond lengths, and hydrogen bonds associated with the Zn(II) ion within the ACE protein molecule were observed, as illustrated in Figure S3.

## 3. Discussion

Numerous studies have been undertaken to investigate the pharmacological characteristics of jellyfish venom [25,26,27,28]. The angiotensin-I-converting enzyme (ACE; EC 3.4.15.1) is considered as one of the essential members in the renin–angiotensin system, playing a crucial physiological role in the regulation of blood pressure [29]. One study has identified that the whole *Nemopilema nomurai* jellyfish has ACE-inhibitory activity, yet it did not identify the key compound(s) from the jellyfish venom [24]. In addition, the ACE-inhibitory peptides were isolated from *Chiropsalmus quadrigatus* Haeckel (box jellyfish) venom hydrolysate [13]. In the present study, we have successfully identified two peptides from *Nemopilema nomurai* jellyfish venom hydrolysate that demonstrate highly significant ACE-inhibitory activity. Therefore, we propose that these isolated peptides have the possibility to be used as candidate molecules for the development of ACE-inhibitor drugs like captopril and enalapril

Various proteases produce polypeptides with distinct compositions and sizes. Enzymatic hydrolysis stands out as the most efficient method for generating bioactive peptides [30,31]. Our results show that the NnV underwent enzyme hydrolysis utilizing two enzymes, protomex and papain. From these, papain was selected as the optimal enzyme based on the degree of hydrolysis. Subsequently, a log-inhibitory analysis was conducted to demonstrate the ACE inhibition of NnV–papain hydrolysate, with captopril utilized as a positive control. The chromatographic purification of proteins is influenced by various factors such as pH, elution volume, gradient characteristics, and ionic strength. The out-standing flow properties of these ion exchangers position them as the primary choice for separating crude protein mixtures during the initial stages of the purification process [32,33]. Peptides with lower molecular weights generally exhibit higher activity compared to those with higher molecular weights, aligning with previous research findings [34,35]. It is postulated that short-chain peptides can adopt a spatial conformation, enabling them to align within the three-dimensional structure of ACE, thereby restricting access for high-molecular-weight peptides [36]. The purification of ACE-inhibitory peptides can be achieved based on their molecular weight, charge, affinity, and polarity [37,38,39]. Various purification methods, including size exclusion chromatography (utilizing Sephadex, Sepharose, Superdex, etc.), ion-exchange chromatography (employing DEAE-cellulose, DE-AE-Sephadex, etc.), and RP-HPLC (utilizing C18, and other columns), are commonly employed, based on the specific characteristics of the peptides. In our study, the enzyme digest NnV-hydrolysate comprised a complex mixture of peptides; it underwent initial separation through ion-exchange chromatography to isolate peptides according to their charges. After the sequential chromatographic separation and ACE-inhibitory evaluation, peak no. 3.2 was identified as a potent candidate, and two peptides were identified using MALDI–TOF/MS. The overall method demonstrated efficient separation, confirming its effectiveness in isolating ACE inhibitors. Hydrophobicity plays a crucial role in facilitating peptide binding to the hydrophobic active site of ACE, consequently enhancing inhibitory activity. Moreover, hydrophobic peptides composed of four to nine amino acids have been observed to passively traverse cell membranes through mechanisms like transcytosis or paracellular diffusion [40,41,42,43]. Thus, applying this hypothesis, the two isolated peptides (IVGRPLANG and IGDEPRHQYL) would have possibly been absorbed, enabling their passage across the intestinal barrier and entry into systemic circulation. The presence of a high concentration of aromatic amino acids and hydrophobic aliphatic amino acids such as isoleucine, leucine, alanine, methionine, and proline are noteworthy, as previous studies [44,45] suggest that these amino acids may enhance ACE-inhibitory activity.

The two identified peptides’ sequences were modeled and docked against an ACE protein molecule, and values were obtained using the HADDOCK score. Hydrogen bond interactions are indispensable for stabilizing the structure of the enzyme–substrate complex, thereby playing a crucial role in facilitating the ACE-catalyzed reaction. Based on the molecular docking, the strong hydrogen interaction suggests that the peptides would effectively inhibit the ACE protein. Despite the absence of a direct interaction be-tween either IVGRPLANG or IGDEPRHQYL and Zn(II), post-docking observations indicate alterations in bond lengths and hydrogen bond formations from their initial states. This outcome implies the potential of the peptides to coordinate with Zn(II) within ACE, potentially distorting its tetrahedral geometry and influencing ACE-inhibitory activities. This observation aligns with other findings reported by Mirzaei et al. (2018) [46] and Wu et al. (2018) [47]. However, this study serves as the primary investigation into drug identification. In future endeavors, we will synthesize the peptides and employ animal models to validate the efficacy of the peptides for further exploration

## 4. Conclusions

In summary, we have identified the angiotensin-converting enzyme inhibitors from *Nemopilema nomurai* jellyfish venom. The major contributions of this study include (i) the papain hydrolysate of NnV showed higher ACE activity; (ii) peak no. 3.2, isolated through sequential chromatography techniques, displayed similar significant ACE activity; (iii) two peptide sequences, IVGRPLANG (896.48) and IGDEPRHQYL (1227.651), have been obtained; (iv) the molecular docking analysis confirms that the binding affinities of the two peptides against ACE molecules are −51.4 ± 2.5 and −62.3 ± 3.3 using the HADDOCK scoring function. Therefore, these findings highlight the pharmacological significance of jellyfish venom-derived compounds for future therapeutic exploration.

## 5. Materials and Methods

### 5.1. Jellyfish Collection and Preparation

Samples of *Nemopilema nomurai* jellyfish were gathered from the Yellow Sea near the Gunsan coast in South Korea. After separating the tentacles, they were promptly placed on ice for further processing. The dissected tentacles underwent cleansing with cold (4 °C) seawater to remove any debris, and the samples were combined with three volumes (*v*/*v*) of chilled fresh seawater. Subsequently, the tentacle-free saltwater was collected and centrifuged at 1000× *g* for 5 min, following 24 h of agitation on a shaker at 4 °C. The resulting nematocyst-rich pellet underwent triple rinsing with fresh seawater. The residual sedimented tentacles underwent additional autolysis overnight at 4 °C, repeating this process for four days, and the nematocyst-rich pellets were once again rinsed with fresh seawater. Ultimately, the nematocyst sample was centrifuged at 500× *g* for 5 min. The resulting pellet (nematocyst) was then freeze-dried and stored at −20 °C for further investigation [48].

### 5.2. Venom Extraction and Preparation

The venom was derived and processed from freeze-dried nematocysts with slight modifications [49]. In summary, venom extraction involved the use of 1 g of nematocyst powder, glass beads (approximately 8000 beads; 0.5 mm in diameter), and 1 mL of ice-cold phosphate-buffered saline (PBS, 0.137 M NaCl, 0.0027 M KCl, 0.01 M Na_2_HPO_4_, 0.0018 M KH_2_PO_4_, pH 7.4) as an extraction buffer. The extraction was carried out through robust vortexing on the mini bead mill at 3000 rpm for 30 s, with this step being repeated more than 10 times at regular intervals while cooling on ice. Subsequently, the venom extract underwent centrifugation for 30 min at 4 °C at 22,000× *g*. The resulting final supernatant was designated as NnV for this study. Protein concentration in the venom was determined using the Bradford test [50] (Bio-Rad, Hercules, CA, USA). Finally, the venom, adjusted according to its protein concentration, was employed for subsequent research. Later, the NnV was employed for dialysis (Fisher brand dialysis tubing with 15 m roll; 25 mm width; 1.98 mL volume; 20 µm thickness; 15.9 mm dry ᴓ; MWCO 12–14 kD), to remove excessive salt for further study.

### 5.3. Enzymatic Hydrolysis of NnV Extract and Assessment of the Degree of Hydrolysis

The hydrolysis of NnV was conducted using protomex and papain, following a slightly modified procedure. The enzyme–substrate reactions were performed with a ratio of 1:100. The reaction conditions, for both papain and protomex, were a pH of 6.8, temperature ranging from 37 to 70 °C, reaction time spanning 0 to 6 h, and concentration range of 0 to 10 µg. To stop the reactions, heat treatment was applied at 90 °C for 15 min. The resulting slurries underwent centrifugation at 3000× *g* for 10 min, and the supernatants were utilized as hydrolysates for subsequent analysis. The Ninhydrin colorimetric method, with slight modifications, was employed to determine the degree of hydrolysis (DH), as described by Huang et al. (2022) [51]. The detection procedure involved taking 0.5 mL of the hydrolysate supernatant post-centrifugation (3000× *g* for 10 min) and combining it with 0.4 mL of sodium phosphate buffer (pH 8.0, 2 M) and 0.4 mL of ninhydrin (2%). Then, the mixture was placed in a 100 °C water bath for 15 min, followed by dilution with distilled water to a total volume of 50 mL after cooling to room temperature. The solution was then allowed to stand for 15 min, and its absorbance was measured at a wavelength of 570 nm. The following equation was used to calculate the degree of hydrolysis:

DH (%) = (H_sample_ − H_blank_)/(H_total_ − H_blank_) × 100

In this context, H_blank_ denotes the absorbance measured with distilled water as the blank. H_total_ represents the absorbance recorded following thorough acid hydrolysis, using 6 M HCl at 120 °C for 24 h.

### 5.4. Assessment of ACE-Inhibitory Activity

The ACE-inhibitory activity was assessed using the ACE kit-WST from Dojindo Laboratories [52], following the assay procedure outlined in the provided technical manual. In summary, a 20-microliter sample solution was introduced to a sample well, as well as to blank 1 and blank 2 wells. Subsequently, 20 microliters of substrate buffer were added to each well. Deionized water was incorporated into the blank 2 well, while 20 mL of enzyme working solution was added to each sample well and blank 1 well. Incubation was carried out at 37 °C for 1 h. Following this, 200 mL of the indicator working solution were introduced to each well, followed by an additional 10 min incubation at room temperature before measuring absorbance at 450 nm using a microplate reader.

ACE inhibition (%) = (A_blank1_ − A_sample_)/(A_blank1_ − A_blank2_) × 100

Here, A_blank1_ represents the absorbance of the control (without ACE inhibition), A_blank2_ corresponds to the absorbance of the reagent alone, and A_sample_ denotes the absorbance of the sample. 

### 5.5. Purification of ACE-Inhibitory Peptides by Sequential Chromatography

The NnV–papain hydrolysate was subjected to ion-exchange chromatography with minor modification, as described by Prakash et al. (2020) [53]. In brief, NnV-hydrolysate was injected into the DEAE Sepharose fast-flow column (1 × 30 cm) (Cytiva), where 20 mM Tris HCl was used as an equilibrium buffer (at pH 6.8). NaCl (0–1 M) in 20 mM Tris HCl buffer was used as an elution buffer at a 0.6 mL/min flow rate by the discontinuous gradient method, and nine peaks were obtained. Each peak’s protein concentration was evaluated using the Bradford Assay. Subsequently, the combined peaks underwent testing for ACE-inhibitory activity, and the most active peak was chosen for further analysis. Peak no. 3 was then subjected to separation using RP-HPLC [54]. The sample was dissolved in 2 mL chromatography water and injected into the ODS-C18 (10 × 250 mm, particle size 5 µm) column. The parameters for elution were 1 mL/min flow, gradient of 0.1% TFA in water (solution A), and gradient in acetonitrile (solution B) as follows: B% as 5, 10, 15, 55, 30, and 5 over time for 0, 5, 10, 20, 25, and 30 min, respectively. UV detection was measured at a wavelength of 214 nm. Then, the peaks were collected and assessed for their ACE-inhibitory activity; potent peaks (peak no. 3.2) were chosen and subjected to MALDI–TOF/MS analysis.

### 5.6. Peptide Identification and Sequencing

The peptides and their sequences were identified through MALDI–TOF/MS at the central facility laboratory in Gyeongsang National University, Republic of Korea. Tests were conducted utilizing Cyano-4-hydroxycinnamic acid (HCCA) as the matrix. The sample (peak no. 3.2), at a volume of of 1 μL, was combined with 1 μL of the matrix, and, subsequently, 2 μL of the resulting mixture was applied onto the target plate. The spectra were obtained using a Bruker auto flex speed MALDI–TOF/MS equipped with a patented smart beam laser, emitting at 355 nm and operating at 200 Hz. Laser-induced fragmentation technology was utilized by initially setting the laser intensity at 30% for the identification of the parent compound, then later adjusted to 45% for fragmentation. The spectra were recorded in reflectron positive ion mode. The de novo sequencing of peptides was determined based on the MS/MS ionic pattern using Bruker Flex analysis version 3.4 [55].

### 5.7. Peptide Structure Prediction Using AlphaFold

The prediction of the 3D structure for the target proteins was executed within the ColabFold platform, using its interface [56]. The protein sequences inputted for AlphaFold modeling were consistent in both instances. In the ColabFold environment, the MMseqs2 method was specifically chosen to generate Multiple Sequence Alignments (MSAs). During this process, the amber relaxation of the model was deactivated, and the prediction for unpaired MSAs was initiated. AlphaFold, employed for predicting the three-dimensional coordinates of protein residues, utilizes pLDDT (predicted pLDDT-Cα) as a metric, ranging from 0 to 100, to indicate the confidence level associated with each prediction. The pLDDT values, reflecting this confidence, are integrated into the B-factor fields of the PDB files. The extraction of these values was facilitated through the utilization of the Bio Python library, version 1.78 [57].

### 5.8. Molecular Docking

The crystal structure of human ACE (ACE) complexed with the inhibitor lisinopril (PDB: 1O8A) was sourced from the RCSB Protein Data Bank (https://www.rcsb.org, accessed on 10 June 2024) and employed as the template for subsequent docking studies. In preparation for docking, the removal of all water molecules and lisinopril inhibitor was performed, while retaining the cofactor zinc atoms within the active site of the ACE model. Polar hydrogens were then added to the ACE model. Molecular docking studies were conducted using the HADDOCK software version 2.4. [46], with the best-ranked docking pose of the purified peptides within the ACE active site determined based on scores and binding energy values. Additionally, PDB sum and PYMOL (visualization tool) was utilized to identify hydrogen bonds, as well as hydrophobic interactions between ACE protein and the identified peptides. The impact of peptides on the Zn(II) tetrahedral geometry was elucidated through the examination of docking results using the LigPlot viewer version 2.2. [58,59].

### 5.9. Statistical Analysis

All the experiments were replicated three times, and the data were subjected to one-way analysis of variance (ANOVA), followed by Dunnett’s test for analysis. Results were expressed as mean ± standard deviation (SD), with significance denoted by * *p* < 0.05, ** *p* < 0.01, and *** *p* < 0.001. Graph Pad prism software was used to conduct all statistical analyses (version 5.1).

## Data Availability

The original contributions presented in the study are included in the article/Supplementary Materials, further inquiries can be directed to the corresponding author/s.

## Supplementary Materials

The following supporting information can be downloaded at: https://www.mdpi.com/article/10.3390/toxins16090410/s1, Figure S1: Prediction of the peptide IVGRPLANG structures by ColabFold; models were ranked based on AlphaFold pTM. Figure S2: Prediction of the peptide IGDEPRHQYL structures by ColabFold; models were ranked based on AlphaFold pTM. Figure S3: The specifics of zinc ion (Zn(II)) coordination with ACE residues are illustrated (A) pre-docking, and post-docking with peptides (B) IVGRPLANG and (C) IGDEPRHQYL. Representation via Ligplot version v.1.4.5 software delineates the formation of hydrogen bonds (indicated by green dotted lines), ligand bonds (represented by blue lines), and non-ligand bonds (depicted by brown lines). Table S1: Peptide structural scoring for the modeled protein using ColabFold and AlphaFold. Table S2: HADDOCK score for IVGRPLANG peptide against ACE protein. Table S3: HADDOCK score for IGDEPRHQYL peptide against ACE protein.

## Abbreviations

NnV—*Nemopilema nomurai* jellyfish venom; ACE—Angiotensin-converting enzyme; ACEI—Angiotensin-converting enzyme inhibitors; HADDOCK—High-ambiguity-driven protein–protein docking; DEAE—Diethylaminomethyl; IEC—Ion-exchange chromatography; RP-HPLC—Reverse-phase high-performance liquid chromatography; MALDI–TOF/MS—Matrix-assisted laser desorption ionization–time of flight/mass spectrometry; pTM—Predicted template modeling score; pLDDT—Predicted local distance difference test; ODS-C18—Octadecyl-silica–Cyclocarbon 18; Zn—Zinc; MWCO—Molecular weight cut-off; DH—Degree of hydrolysis; HCCA—Cyano-4-hydroxycinnamic acid; MSA—Multiple sequence alignment; PyMOL—Cross-platform molecular graphic tool; RCSB—Research Collaboratory for Structural Bioinformatics; PDB—Protein data bank; SD—Standard deviation; ANOVA—Analysis of variance.
