# Supplementary material for: Identification of New Angiotensin-Converting Enzyme Inhibitory Peptides Isolated from the Hydrolysate of the Venom of Nemopilema nomurai Jellyfish
Source: Toxins (Basel). 2024 Sep 20;16(9):410. doi: 10.3390/toxins16090410 (PMC11435582; doi:10.3390/toxins16090410)
Supplement: Supplementary file 1 [file toxins-16-00410-s001.zip › toxins-3131958-supplementary.pdf]

Supplementary Materials: Identification of New Angiotensin-Converting Enzyme Inhibitory Peptides Isolated from the Hydrolysate of the Venom of Nemopilema nomurai Jellyfish

Ramachandran Loganathan Mohan Prakash, Deva Asirvatham Ravi, Du Hyeon Hwang, Changkeun Kang, and Euikyung Kim

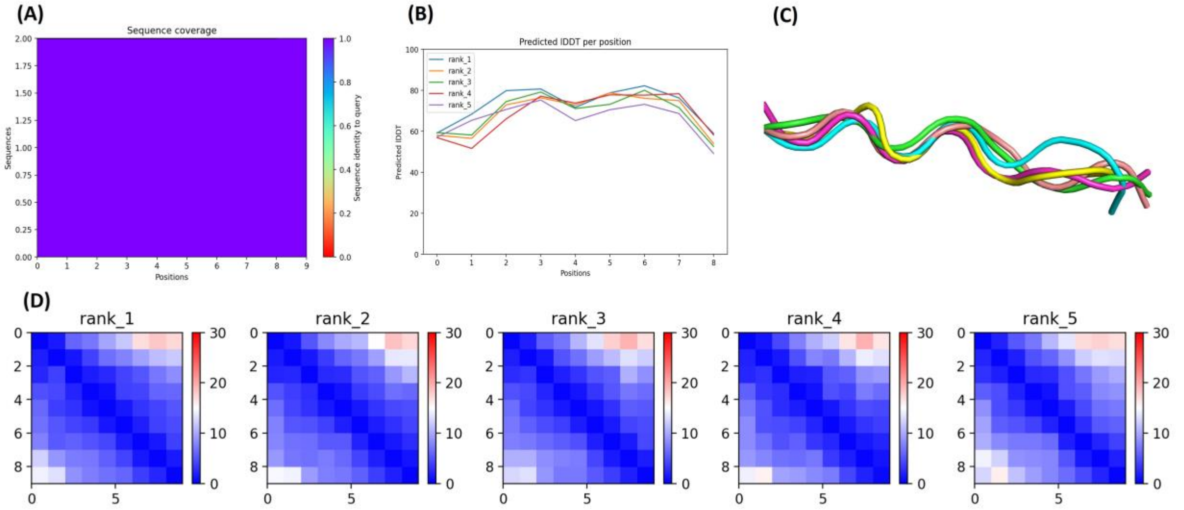

Figure S1. Prediction of the peptide IVGRPLANG structures by ColabFold and models were ranked based on AlphaFold pTM Score.

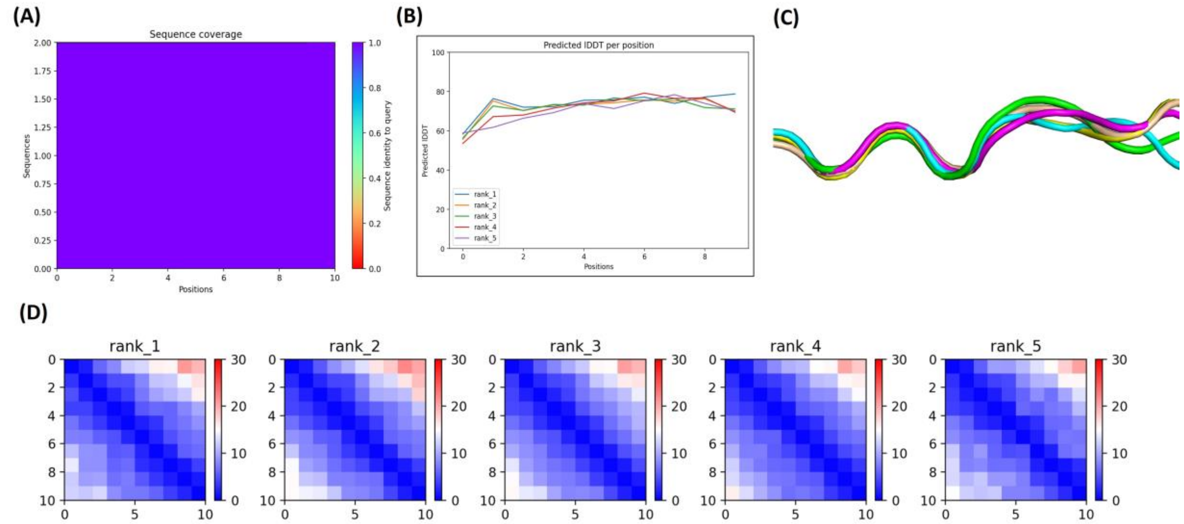

Figure S2. Prediction of the peptide IGDEPRHQYL structures by ColabFold and models were ranked based on AlphaFold pTM Score.

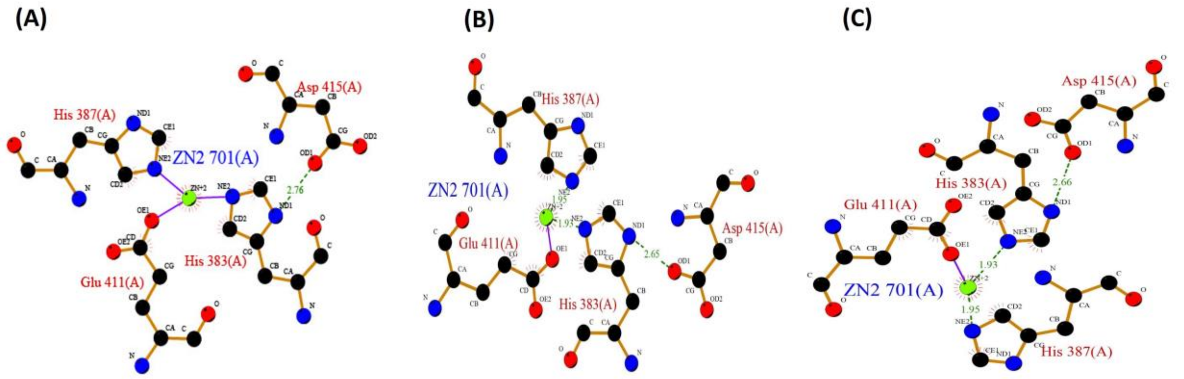

Figure S3. The specifics of zinc ion (Zn (II)) coordination with ACE residues are illustrated both (A) pre-docking and post-docking with peptides (B) IVGRPLANG and (C) IGDEPRHQYL. Representation via Ligplot version v.1.4.5 software delineates the formation of hydrogen bonds (indicated by green dotted lines), ligand bonds (represented by blue lines), and non-ligand bonds (depicted by brown lines).

Table S1. Peptide Structural Scoring for modeled protein using ColabFold and AlphaFold

Peptide sequence	Score	Model 1	Model 2	Model 3	Model 4	Model 5
IVGRPLANG	pLDDT	68.6	69	68.9	72.9	66.2
	pTM	0.0466	0.0458	0.0453	0.0461	0.0443
IGDEPRHQYL	pLDDT	70	71.2	73.8	71.8	72
	pTM	0.0412	0.0402	0.04	0.0394	0.0396

Table S2. HADDOCK score for IVGRPLANG peptide against ACE protein

	**Cluster 1**	**Cluster 2**	**Cluster 3**	**Cluster 4**	**Cluster 5**	**Cluster 6**	**Cluster 7**	**Cluster 8**	**Cluster 9**	**Cluster 10**
HADDOCK score	-25.1 +/- 1.6	-36.9 +/- 4.4	-34.8 +/- 1.8	-25.1 +/- 1.4	-51.4 +/- 2.5	-30.4 +/- 2.0	-25.3 +/- 4.1	-25.3 +/- 6.9	-34.1 +/- 5.6	-33.2 +/- 1.3
Cluster size	16	12	10	9	8	6	5	5	5	5
RMSD from the overall lowest-energy structure	1.7 +/- 0.0	2.7 +/- 0.2	4.0 +/- 0.1	1.6 +/- 0.1	0.2 +/- 0.1	3.2 +/- 0.1	1.0 +/- 0.0	2.0 +/- 0.0	1.4 +/- 0.1	2.1 +/- 0.1
Van der Waals energy	-29.5 +/- 2.8	-30.0 +/- 7.7	-35.8 +/- 4.2	-35.9 +/- 4.2	-43.7 +/- 6.0	-40.2 +/- 2.9	-35.1 +/- 4.9	-32.3 +/- 5.5	-36.1 +/- 2.4	-32.3 +/- 6.0
Electrostatic energy	-66.3 +/- 14.9	-141.5 +/- 15.1	-89.7 +/- 38.0	-52.0 +/- 8.1	-110.7 +/- 13.9	-80.3 +/- 15.0	-70.6 +/- 19.1	-50.1 +/- 14.5	-48.2 +/- 48.1	-110.8 +/- 37.5
Desolvation energy	-1.1 +/- 1.1	6.6 +/- 1.7	5.9 +/- 0.8	-0.7 +/- 3.0	-1.1 +/- 2.5	5.5 +/- 0.8	-1.2 +/- 2.1	0.3 +/- 1.3	-4.0 +/- 2.5	2.0 +/- 1.5
Restraints violation energy	187.2 +/- 39.7	148.7 +/- 67.0	130.2 +/- 24.9	219.5 +/- 23.8	155.3 +/- 56.4	203.3 +/- 55.6	250.9 +/- 62.9	166.7 +/- 21.2	156.6 +/- 42.9	192.8 +/- 22.0
Buried Surface Area	922.5 +/- 16.2	1018.4 +/- 79.8	972.9 +/- 58.6	1137.7 +/- 31.6	1331.2 +/- 75.9	1118.6 +/- 47.4	1155.5 +/- 112.2	926.8 +/- 103.9	1165.4 +/- 12.8	993.5 +/- 41.6
Z-Score	0.9	-0.6	-0.3	0.9	-2.5	0.2	0.9	0.9	-0.3	-0.1

Table S3. HADDOCK score for IGDEPRHQYL peptide against ACE protein

	**Cluster 1**	**Cluster 2**	**Cluster 3**	**Cluster 4**	**Cluster 5**	**Cluster 6**	**Cluster 7**	**Cluster 8**	**Cluster 9**	**Cluster 10**
HADDOCK score	-59.2 +/- 1.9	-58.7 +/- 3.2	-47.4 +/- 3.6	-43.1 +/- 10.6	-40.2 +/- 7.6	-59.0 +/- 8.7	-62.3 +/- 3.3	-41.8 +/- 2.3	-40.0 +/- 5.2	-50.2 +/- 8.0
Cluster size	28	16	14	7	6	6	6	5	4	4
RMSD from the overall lowest-energy structure	0.8 +/- 0.1	2.7 +/- 0.0	2.5 +/- 0.1	2.6 +/- 0.1	2.2 +/- 0.0	0.3 +/- 0.2	2.2 +/- 0.0	3.3 +/- 0.0	2.3 +/- 0.0	2.1 +/- 0.0
Van der Waals energy	-39.5 +/- 4.3	-44.1 +/- 4.2	-48.2 +/- 2.9	-32.2 +/- 6.5	-25.8 +/- 2.2	-38.7 +/- 7.9	-46.2 +/- 2.9	-31.9 +/- 0.9	-34.7 +/- 2.7	-36.4 +/- 3.1
Electrostatic energy	-168.2 +/- 28.1	-150.7 +/- 10.1	-66.7 +/- 21.6	-137.7 +/- 23.9	-176.6 +/- 23.2	-181.4 +/- 19.5	-176.8 +/- 22.7	-183.5 +/- 1.4	-133.0 +/- 19.1	-145.3 +/- 22.2
Desolvation energy	-4.3 +/- 2.2	-7.1 +/- 1.2	-12.4 +/- 4.2	-6.1 +/- 2.1	4.7 +/- 3.1	1.4 +/- 2.2	1.8 +/- 1.5	-5.7 +/- 2.5	-4.0 +/- 1.3	-8.7 +/- 1.3
Restraints violation energy	182.3 +/- 49.0	226.3 +/- 24.9	265.5 +/- 23.6	227.6 +/- 27.6	162.2 +/- 44.3	145.4 +/- 40.5	174.4 +/- 25.7	324.5 +/- 37.0	254.1 +/- 61.3	239.2 +/- 9.4
Buried Surface Area	1140.7 +/- 58.9	1225.9 +/- 59.0	1295.6 +/- 69.5	1125.9 +/- 83.8	983.4 +/- 74.8	1087.6 +/- 162.1	1202.6 +/- 46.9	1154.6 +/- 59.0	999.7 +/- 41.9	1224.2 +/- 63.6
Z-Score	-1.1	-1	0.3	0.8	1.2	-1	-1.4	1	1.2	0
